# Reforming Medical Education in India: Beyond Marks as the Sole Determinant of Postgraduate Seat Allocation

**DOI:** 10.7759/cureus.104849

**Published:** 2026-03-08

**Authors:** Arpit Suman, Tej P Gupta, Sanjay Rai

**Affiliations:** 1 Preventive Medicine, Shri Ram Murti Smarak Institute of Medical Sciences, Bareilly, IND; 2 Orthopaedics, Base Hospital Delhi Cantonment, Delhi, IND; 3 Orthopaedics, Shri Ram Murti Smarak Institute of Medical Sciences, Bareilly, IND

**Keywords:** competency-based medical education, medical education, medical education in india, national medical commission (nmc), postgraduate

## Abstract

This editorial examines the growing concerns surrounding postgraduate (PG) seat allocation in India amid recent regulatory reforms and the rapid expansion of training capacity. Drawing on policy developments between 2023 and 2025, including centralised online counselling and significant increases in PG seats, it analyses the continued reliance on a single high-stakes entrance examination as the primary determinant of specialist training opportunities. The recent lowering of qualifying percentiles to fill vacant seats has intensified debate regarding academic standards, merit, and workforce preparedness. This discussion highlights the tension between quantitative expansion and qualitative assurance in medical education. The editorial contributes to the national discourse by underscoring the misalignment between competency-based undergraduate reforms and marks-driven postgraduate selection, and advocates for a structured, multi-dimensional assessment framework that better reflects clinical competence, professionalism, and healthcare system needs.

## Editorial

India’s medical education system stands at a critical juncture. Over the past few years, regulatory restructuring, expansion of training capacity, and national entrance examinations have reshaped the trajectory of medical careers. The National Medical Commission (NMC) introduced the Post-Graduate Medical Education Regulations, 2023, mandating common online counselling based on National Eligibility cum Entrance Test for Postgraduate (NEET-PG) merit lists and eliminating independent institutional admissions, thereby further centralising the allocation process [1].

Despite these efforts to standardise and streamline admissions, the reliance on a single high-stakes exam (NEET-PG) remains entrenched. Recent controversies highlight the system’s fragility: for the 2025-26 PG admissions cycle, the National Board of Examinations in Medical Sciences (NBEMS) controversially lowered NEET-PG qualifying percentiles drastically (e.g., general category from 50th to 7th percentile, with reserved categories reaching 0th percentile), allowing candidates with very low or even negative scaled scores to enter counselling in an attempt to fill vacant seats [2,3]. Critics from major medical bodies described this as “unprecedented and illogical,” warning that it threatens academic quality and future healthcare standards [4].

At the same time, the government continues to expand capacity: over 29,000 postgraduate seats have been added between 2020-21 and 2025-26, and an additional 10,023 seats were approved under central schemes to strengthen medical education infrastructure nationwide [5]. This quantitative growth demonstrates commitment to workforce expansion but also highlights a widening gap between seat availability and mechanisms for assessing candidate readiness.

The evolution of Competency-Based Medical Education (CBME) illustrates another dimension of ongoing reform. Although the NMC released updated CBME Curriculum Guidelines for 2024 to modernise training objectives and prioritise clinical competencies, these guidelines were withdrawn amid controversy, with a revised framework promised to address stakeholder concerns [6]. The simultaneous push for competency-driven curricula at the undergraduate level and the persistence of marks-driven postgraduate selection underscores structural incongruities in the education pathway.

Globally, postgraduate selection systems increasingly incorporate multi-factorial assessment tools - structured interviews, situational judgement tests, portfolios, and documented clinical performance - to predict future professional success, complementing cognitive scores [7]. India’s current exam-centric model fails to integrate such broader metrics, narrowing the definition of merit to test performance alone.

There are equity implications as well. Overreliance on a single exam advantageously positions candidates with access to extensive coaching and resources, exacerbating socioeconomic disparities in specialist training access.

Reform does not imply abandoning centralised benchmarking entirely. Rather, a hybrid selection model that retains standardised testing while incorporating longitudinal academic performance, structured clinical assessments, and professional behaviours could provide a richer, fairer basis for allocating PG seats. Structural recalibration aligning with NMC’s regulatory trajectory and workforce needs would enhance both educational quality and service outcomes.

India has demonstrated significant ambition in reforming medical education. The next imperative is aligning postgraduate selection processes with evolving regulatory frameworks and educational principles, ensuring that merit encompasses not just marks on a paper, but also the holistic competencies requisite for high-quality patient care.
